# Supplementary material for: Neural computations in the foveal and peripheral visual fields during active search
Source: eLife. 2026 Aug 20;15:RP109498. doi: 10.7554/eLife.109498 (PMC13493125; doi:10.7554/eLife.109498)
Supplement: Peer review history

# Editor report: eLife Assessment

**DOI:** 10.7554/eLife.109498.3.sa0

**Authors:** Tirin Moore

**Affiliations:** Stanford University, Howard Hughes Medical Institute United States

**Keywords:** Important, Solid

This study reports **important** and invaluable findings that advance understanding of how attention is distributed between what we look at directly and what lies outside the center of gaze during active visual search. The evidence supporting the main claims is **solid**, with a large and rich dataset spanning multiple brain areas, although some aspects of the interpretation would benefit from additional controls and clearer separation of attention from eye-movement planning. The work will be of particular interest to researchers studying attention, visual perception, and eye movements behavior.

# Referee report: Reviewer #2 (Public review)

**DOI:** 10.7554/eLife.109498.3.sa1

Summary:

In natural visual behavior, such as when one is looking for a face in the crowd, the eyes are moved from site to site, seeking possible matching targets. This involves attention both to the current view at center of vision (the foveal location) as well as to upcoming views via attention to targets in the periphery. While it has been established that attention generally enhances neuronal response (compared to simple visual activation) at the attended spatial location, this study provides solid evidence that attention during active visual search leads to neuronal response enhancement only when the eye moves towards targets that exhibit the desired feature and category. This study thus moves the field towards understanding the neural encoding of active vision.

This study examines the neuronal basis of feature selective attention during active, freely behaving visual search. Traditional electrophysiological studies on visual attention in monkeys commonly used an eye fixation with covert attention paradigm, but have not sufficiently addressed the roles of both foveal and peripheral attention in play during natural looking behavior. Here, the authors present a novel paradigm in which, during eye movement mediated search neuronal receptive fields are recorded in multiple cortical areas (sensory V4, temporal and prefrontal areas). In this manner, as the eye foveates, items in the array fall into foveal or non-foveal recorded sites. Thus, the experimental paradigm is elegant, offering the opportunity to make multiple types of comparisons: target/distractor, towards/away from fovea, areal. Specifically, following a category cue (face, house, hand, flower), freely initiated saccades are made to locate a categorically matching 'target' in an array of distractors. Feature attention is assessed by comparing eye saccades made to targets vs to distractors. Spatial attention is assessed by comparing saccades made 'towards' vs 'away' from targets. Statistics are rigorous and nicely designed. Detailed association of simultaneously obtained eye movement sequences and neural parameters are well done. These are valuable data which will contribute to our understanding of attentional modulation in visual search.

The significance of these findings is fundamental. Decades of attention research in vision have been based on the paradigm of visual fixation and covert peripheral attention. However, increasingly the field has moved towards understanding how the visual system works during active vision. Here, the authors use an active visual search paradigm and record from key mid-tier (V4) and higher order (IT, PFC) areas. They find enhancement of attention both in the foveal and peripheral locations, and, furthermore, marked by a high degree of feature and categorical specificity. That is, while attention generally enhances neuronal response (compared to simple visual activation) at the attended spatial location, this study provides solid evidence that attention during active visual search leads to neuronal response enhancement only when the eye moves towards targets that exhibit the desired feature and category. This provides valuable data for the concept of a foveal-peripheral spatiotemporal attentional window in natural vision. The controls (comparisons of neuronal response during looks to targets vs distractors and looks towards and away from the target) and statistical rigor make these findings compelling. There will likely be additional future impacts of this study. For example, the eye movement patterns collected in this study may also provide a valuable dataset for future study of understanding search strategies. Goal-directed vs non-goal-directed task comparisons could be designed to test possible circuit models. Although much remains unknown regarding how and where frontal and temporal signals are integrated during active search, these data contribute important guideposts for future models of active visual search.

# Referee report: Reviewer #3 (Public review)

**DOI:** 10.7554/eLife.109498.3.sa2

In this manuscript, the authors investigate the role of attention in foveal processing during a naturalistic task. They record neural activity from extrastriate visual areas V4 and inferotemporal cortex, as well as from the lateral prefrontal cortex, in macaques performing a free-gaze visual search task. In this task, animals searched for a face or house target among multiple complex stimuli, with no constraints on eye movements. Unlike classic studies of visual attention, which often rely on controlled fixation, this work examines neural activity in both foveal and peripheral receptive fields during naturalistic eye movements.

The main question addressed by the authors is how feature-based attention is distributed and coordinated across foveal and peripheral visual fields during active search, and how this attentional processing influences saccade behavior. The authors show that foveal units in visual areas exhibit feature-based attentional enhancement, with stronger responses when a fixated stimulus is a target compared to when the same stimulus serves as a distractor. Peripheral units in visual and prefrontal areas show both feature-based and spatial attentional modulation, consistent with prior work. Finally, the authors show that attentional modulation depends primarily on stimulus category rather than response magnitude, with neurons showing similar enhancement for all images within the target category regardless of how strongly individual images drive the cell.

There are several notable strengths of this paper including:

(1) Disentangling feature-based and spatial attention during naturalistic vision remains a central challenge. This paper tackles both simultaneously, parsing neural populations by object selectivity (face-selective, house-selective, non-selective) and RF position (foveal vs. peripheral).

(2) The unconstrained search task (Fig. 1A) moves beyond the dominant fixed-gaze, cued-attention designs (Zhou & Desimone, 2011) to study attention as it operates during natural behavior, with sequential fixations and voluntary saccades.

(3) The scale of the multi-area recordings is a major strength and is well aligned with current trends in primate and human neuroscience toward large-scale, multi-area recordings. Simultaneous recordings from visual and prefrontal areas, comprising over 4,900 foveal units and more than 1,500 peripheral units, enable meaningful cross-area latency comparisons and area-specific analyses of attentional modulation. This study builds on the authors' previous analyses of this dataset by expanding the scope to show that feature-based attention generalizes across neuronal classes and operates on categorical identity rather than response magnitude.

(4) The combination of simultaneous multi-area recordings and a rich behavioral paradigm provide a dataset that is well suited for population decoding, cross-area interaction analyses, and trial-by-trial prediction of saccade choices, which could substantially deepen mechanistic understanding beyond the largely univariate comparisons presented here.

While the data broadly support the paper's main conclusions, several issues limit the strength of the mechanistic interpretation and should be taken into consideration:

(1) Receptive field size is not explicitly quantified and may confound foveal-peripheral comparisons. Units are classified as foveal or peripheral based on responsiveness to the cue versus the search array (Methods, p. 17), but the manuscript lacks essential information about receptive field sizes, eccentricities, and the number of search stimuli falling within each receptive field and related proper controls. This is critical because receptive fields in visual area V4 at foveal eccentricities are relatively small (Gattass et al., 1988; Desimone & Schein, 1987), whereas receptive fields in inferotemporal cortex can span several degrees to tens of degrees and often include the fovea (Op de Beeck & Vogels, 2000; DiCarlo & Maunsell, 2003; Zoccolan et al., 2007). Given the 2{degree sign} × 2{degree sign} stimulus size, multiple search items could potentially fall simultaneously within peripheral receptive fields. This introduces a potential confound, as attentional modulation is known to be strongest when multiple stimuli appear within a single receptive field (Reynolds et al., 1999). Although the authors acknowledge this issue for visual area V4 (p. 17), it is neither quantified nor controlled for. Without explicit receptive field mapping relative to the search array, comparisons between foveal and peripheral units, as well as between visual areas, are difficult to interpret cleanly.

(2) Attentional modulation is difficult to dissociate from saccade planning and decision-related signals. The free-gaze paradigm enhances ecological validity but introduces a temporal confound: mean distractor fixation durations are approximately 156 ms (p. 9), while attentional effects emerge between 137 and 170 ms after fixation onset (Fig. 2). As a result, the reported attentional modulation coincides with preparation of the subsequent saccade. Neural activity measured in the primary analysis window (150-225 ms; p. 19) therefore likely reflects a mixture of visual, attentional, motor planning, target recognition, and behavioral relevance signals, all of which are known to modulate responses in visual areas at similar latencies (e.g., Chelazzi et al., 1998). Moreover, target fixations (~257 ms) and distractor fixations (~156 ms) occur on fundamentally different behavioral timescales, which may inflate apparent foveal attentional effects. While the authors suggest that these timing differences support the idea that foveal feature-based attention facilitates prolonged fixation on target stimuli, this interpretation is not fully supported by the current analyses. That said, the saccade-aligned analyses of peripheral units (Fig. S3) partially mitigate this concern by demonstrating that feature-based modulation persists through saccade execution.

(3) The "attention-out" condition for spatial attention lacks directional control. In the spatial attention analyses (Fig. 4D-F), the "attention-out" condition appears to include all fixations followed by saccades directed away from the receptive field, regardless of saccade direction. This differs from classic spatial attention designs, which typically use controlled anti-saccades or saccades to fixed locations opposite the receptive field (e.g., Moore & Armstrong, 2003; Gregoriou et al., 2009). Saccades directed toward locations adjacent to, but outside, the receptive field may still partially engage spatial attention mechanisms near the receptive field via broad attentional fields or motor preparation gradients (Bisley & Goldberg, 2010). In addition, the "attention-out" condition likely contains a heterogeneous mixture of trials in which the stimulus in the receptive field is either a target or a distractor, since feature-based attention effects are derived from this same pool of trials. As a result, spatial and feature attention effects are not fully orthogonal, and variance related to feature attention may already be embedded in the spatial attention baseline.

[Editors' note: the authors have provided responses to each of these points.]

# Author comment: Author response

**DOI:** 10.7554/eLife.109498.3.sa3

**Authors:** Jie Zhang, Xiaocang Zhu, Zhengyu Ma, Shanshan Wang, Yutian Wang, Hossein Esteky, Yonghong Tian, Huihui Zhou

**Affiliations:** Shenzhen Institutes of Advanced Technology Shenzhen China; Shenzhen Institutes of Advanced Technology Shenzhen China; Peng Cheng Laboratory Shenzhen China; Shenzhen Institutes of Advanced Technology Shenzhen China; Shenzhen University of Advanced Technology Shenzhen China; School of Medicine, Shaheed Beheshti University Tehran Iran; Peng Cheng Laboratory Shenzhen China; Peng Cheng Laboratory Shenzhen China

The following is the authors’ response to the original reviews.

**Public Reviews:**

**Reviewer #1 (Public review):**

Summary:

This manuscript aims to differentiate between foveal and peripheral attentional mechanisms in visual and frontal brain regions in monkeys engaged in a free-gaze visual search task.

Strengths:

The manuscript is clearly written, the question is important, and the behavioral task is interesting.

Weaknesses:

I have two major concerns.

(1) The authors interpret divergence in neural responses to target vs nontarget as attention. But it is not. The subject has to attend to both target and nontarget stimuli to determine the stimulus category and thereby decide on the next action. Thus, divergence between target and nontarget responses could reflect categorical discrimination, but I am not sure this can be interpreted as attentional modulation. While it may be tempting to suggest that finding a stimulus of a specific category is "feature attention", analogous to, e.g., attending to the red stimulus, I don't believe this is correct. For the former, the animals have to attend to a stimulus, and examine the stimulus to determine the stimulus category, unlike a simpler discrimination, which may pop out. Given this, I am unconvinced that the interpretations in this manuscript are valid.

We thank the reviewer for raising this concern. Selective attention is a process of focusing on goal-relevant stimuli (targets) while ignoring irrelevant distractions. Importantly, attentional selection is not limited to simple visual features (e.g., color, shape, or motion); it can also operate over more complex features. For example, objects themselves can serve as units of attentional selection [1, 2], and feature-based attentional effects have been observed when searching for images that match the cued images or image patches [3, 4]. In this context, attention can be directed either to overall features of an object or to objects as configurations of multiple non-spatial features. Furthermore, attention to the category of stimuli has been extensively investigated in fMRI experiments in humans [5-8], and it has been shown that attention can warp the representations of semantically related categories when participants search for different categories [7].

Similarly, in our study, monkeys were trained to search for images that matched the category of the cue. The neural responses to targets versus distractors were compared while constrained to the same stimuli across different trials, ensuring that the observed response divergence was not due to the physical category of the targets and distractors. We also included only neural responses occurring prior to fixations associated with target selection, that is, before the monkeys made a behavioral choice, thereby controlling for potential contributions of target detection or decision-related signals to the observed effects.

We have clarified and addressed this point in the Discussion as follows:

“Feature-based attention to simple visual features such as color, shape, or motion has been extensively studied [1, 3, 5, 7-9, 11, 12, 64]. Attention can also operate over more complex features. For example, objects themselves can serve as units of attentional selection [65, 66], and feature-based attentional effects have been observed when searching for images that match the cued images or image patches [6, 67]. In this context, attention can be directed either to overall features of an object or to objects as configurations of multiple non-spatial features. Furthermore, attention to the category of stimuli has been extensively investigated in fMRI experiments in humans [68-71], and it has been shown that attention can warp the representations of semantically related categories when participants search for different categories [70]. In this study, the neural responses to targets versus distractors were compared while constrained to the same stimuli across different trials, ensuring that the observed response divergence was not due to the physical category of the targets and distractors.”

(2) Regarding the RF classification of foveal and peripheral RFs for IT and PFC, prior work suggests that neurons in IT cortex (especially AIT) and PFC have RFs that largely include the foveal visual field. So, it would be important to include figures that show the RFs of neurons classified as foveal versus peripheral for all three areas.

We thank the reviewer for raising this important point. We agree with the reviewer that neurons in IT cortex and PFC often have RFs that include the foveal visual field. We did record foveal units with both focal and broad foveal RFs; however, in our analysis we only included neurons with focal foveal RFs to exclude the influence of peripheral stimuli. We defined focal foveal-RF units as those that responded solely to the cue in the foveal region and not to items in the search array presented at least 5° away from the central fixation point, ensuring that their RFs did not extend to these peripheral locations. The items were also separated by at least 5° from each other, excluding the possibility that peripheral stimuli fell within their RFs during fixations. By definition, their RFs were restricted to the central point. This is further supported by Fig. S1A-H, which shows no responses to items in the search array at peripheral locations. We have made modifications in the Results and Methods as follows:

“Notably, the items in the search array were presented at least 5° from the central fixation point and were also separated by at least 5° from each other, excluding the possibility that peripheral stimuli fell within their foveal RFs during fixations.”

And:

“In this study, our focus was on units with focal foveal RFs and units with localized peripheral RFs. All further analyses were conducted on these units.”

We modified Fig. 1 to illustrate the RFs of neurons classified as peripheral, which were also characterized in our previous study using the same dataset [9]. The peripheral population exhibits no responses to the central cue (Fig. S1I–T).

**Reviewer #2 (Public review):**

Summary:

In natural visual behavior, such as when one is looking for a face in the crowd, the eyes are moved from site to site, seeking possible matching targets. This involves attention both to the current view at the center of vision (the foveal location) as well as to upcoming views via attention to targets in the periphery. While it has been established that attention generally enhances neuronal response (compared to simple visual activation) at the attended spatial location, this study provides solid evidence that attention during active visual search leads to neuronal response enhancement only when the eye moves towards targets that exhibit the desired feature and category. This study thus moves the field towards understanding the neural encoding of active vision.

This study examines the neuronal basis of feature-selective attention during active, freely behaving visual search. Traditional electrophysiological studies on visual attention in monkeys commonly used an eye fixation with a covert attention paradigm, but have not sufficiently addressed the roles of both foveal and peripheral attention in play during natural looking behavior. Here, the authors present a novel paradigm in which, during eye-movement mediated search, neuronal receptive fields are recorded in multiple cortical areas (sensory V4, temporal, and prefrontal areas). In this manner, as the eye foveates, items in the array fall into foveal or non-foveal recorded sites. Thus, the experimental paradigm is elegant, offering the opportunity to make multiple types of comparisons: target/distractor, towards/away from fovea, and areal. Specifically, following a category cue (face, house, hand, flower), freely initiated saccades are made to locate a categorically matching 'target' in an array of distractors. Feature attention is assessed by comparing eye saccades made to targets vs to distractors. Spatial attention is assessed by comparing saccades made 'towards' vs 'away' from targets. Statistics are rigorous and nicely designed. The detailed association of simultaneously obtained eye movement sequences and neural parameters is well done. These are valuable data that will contribute to our understanding of attentional modulation in visual search.

Strengths:

The significance of these findings is fundamental. Decades of attention research in vision have been based on the paradigm of visual fixation and covert peripheral attention. However, increasingly, the field has moved towards understanding how the visual system works during active vision. Here, the authors use an active visual search paradigm and record from multiple areas (V4, IT, PFC). They find enhancement of attention both in the foveal and peripheral locations, and, furthermore, a high degree of feature and categorical specificity. This provides valuable data for the concept of a foveal-peripheral attentional window in natural vision. The controls (comparisons of neuronal response during looks to targets vs distractors, and looks towards and away from the target) and statistical rigor make these findings quite compelling.

Weaknesses:

While the study is generally quite strong, there are a few weaknesses to be addressed.

(1) Little rationale is provided for recording in the selected areas, V4, IT, and PFC. Given the respective roles in sensory, object recognition, and goal-directed behavior, some rationale for this design should be offered, and commonalities/distinctions between these areas should be discussed.

We thank the reviewer for the suggestion and we modified and added the rationale to the Introduction as follows:

“V4 and inferotemporal cortex (IT), as the middle and high-level areas of the ventral visual stream, are important for object recognition and categorization [27-34], and their roles have been extensively studied in central vision. At the neuronal level, however, most investigations have largely neglected their functions during active, free-gaze visual search. The prefrontal cortex, including LPFC, has long been implicated as a source of top-down signals that bias the selection of attended features and modulate visual cortical responses [6, 9, 11, 35-40]. Although target-related visual responses have been reported in IT during visual exploration [41], and target-selective responses have been observed in the human medial temporal lobe (MTL) [42] and medial frontal cortex (MFC) [43] during visual search, these studies did not map the receptive fields (RFs) of recorded neurons.”

We also added a discussion as follows:

“Some studies have provided evidence for integration between peripheral and foveal feature information across saccades, including features such as stimulus color [58, 59] and object orientation [60, 61], and visual features have been shown to be predictively remapped prior to saccades [62]. Our finding provides a potential neuronal mechanism that may support this integration process [63]. We found that LPFC’s extensive representation of the visual periphery provides a neural substrate for monitoring the broader search array. Crucially, our finding that LPFC activity temporally precedes attentional effects in the visual area—consistent with previous studies [6, 9, 11, 35-40] suggests that it does not merely reflect peripheral sensory input. Instead, LPFC likely acts as a top-down orchestrator, projecting task-relevant templates derived from current foveal goals onto peripheral candidate locations, a possibility that warrants further investigation.”

(2) Given the reliance of all analyses on saccadic behavior (towards target/distractor, towards/away from target), additional description and summaries of eye movement behavior during single trials and across trials should be provided.

We thank the reviewer for this helpful suggestion. We have added a description of saccade behavior to the Results as follows:

“The mean number of saccades monkeys made to find the target after the onset of the search array was 2.25 ± 1.35 (mean ± SD across trials; Table 1) of correct trials, and the mean saccade amplitude was 7.99° ± 3.58° (mean ± SD across saccades; Table 1). Monkeys could fixate on each distractor or the target freely, provided they did not maintain fixation on the target for longer than 800 ms. Across sessions, 42.44% ± 3.6% of saccades were directed to distractors, 57.56% ± 3.6% to targets, and 12.59% ± 3.46% were saccades away from targets (see our previous studies [44-46] for detailed behavioral analyses).”

We have modified Fig. 1A and its legend to illustrate the saccadic patterns of monkeys during the search task.

We have also included Table 1, which summarizes eye movement behaviors.

(3) The dependency of findings on top-down (categorical & feature-specific) task design should be discussed.

We thank the reviewer for the suggestion and added a discussion as follows:

“In this task, attention is strongly guided by top-down goals, which bias processing toward behaviorally relevant features and object categories [2, 50, 51]. Top-down attention, including categorical and feature-specific components, has been shown to modulate neural processing across the visual pathway based on task demands and to originate from distributed frontoparietal control networks [11, 35-38, 40]. Our study provides further insight into the mechanisms of goal-directed visual attention, as it is among the first to demonstrate foveal feature attention effects during free-gaze visual search, as well as the distribution of feature and spatial attention across the entire visual field.”

**Reviewer #3 (Public review):**

In this manuscript, the authors investigate the role of attention in foveal processing during a naturalistic task. They record neural activity from extrastriate visual areas V4 and inferotemporal cortex, as well as from the lateral prefrontal cortex, in macaques performing a free-gaze visual search task. In this task, animals searched for a face or house target among multiple complex stimuli, with no constraints on eye movements. Unlike classic studies of visual attention, which often rely on controlled fixation, this work examines neural activity in both foveal and peripheral receptive fields during naturalistic eye movements.

The main question addressed by the authors is how feature-based attention is distributed and coordinated across foveal and peripheral visual fields during active search, and how this attentional processing influences saccade behavior. The authors show that foveal units in visual areas exhibit feature-based attentional enhancement, with stronger responses when a fixated stimulus is a target compared to when the same stimulus serves as a distractor. Peripheral units in visual and prefrontal areas show both feature-based and spatial attentional modulation, consistent with prior work. Finally, the authors show that attentional modulation depends primarily on stimulus category rather than response magnitude, with neurons showing similar enhancement for all images within the target category regardless of how strongly individual images drive the cell.

There are several notable strengths of this paper, including:

(1) Disentangling feature-based and spatial attention during naturalistic vision remains a central challenge. This paper tackles both simultaneously, parsing neural populations by object selectivity (face-selective, house-selective, non-selective) and RF position (foveal vs. peripheral).

(2) The unconstrained search task (Figure 1A) moves beyond the dominant fixed-gaze, cued-attention designs (Zhou & Desimone, 2011) to study attention as it operates during natural behavior, with sequential fixations and voluntary saccades.

(3) The scale of the multi-area recordings is a major strength and is well aligned with current trends in primate and human neuroscience toward large-scale, multi-area recordings. Simultaneous recordings from visual and prefrontal areas, comprising over 4,900 foveal units and more than 1,500 peripheral units, enable meaningful cross-area latency comparisons and area-specific analyses of attentional modulation. This study builds on the authors' previous analyses of this dataset by expanding the scope to show that feature-based attention generalizes across neuronal classes and operates on categorical identity rather than response magnitude.

(4) The combination of simultaneous multi-area recordings and a rich behavioral paradigm provides a dataset that is well-suited for population decoding, cross-area interaction analyses, and trial-by-trial prediction of saccade choices, which could substantially deepen mechanistic understanding beyond the largely univariate comparisons presented here.

While the data broadly support the paper's main conclusions, several issues limit the strength of the mechanistic interpretation and should be taken into consideration:

(1) Receptive field size is not explicitly quantified and may confound foveal-peripheral comparisons. Units are classified as foveal or peripheral based on responsiveness to the cue versus the search array (Methods, p. 17), but the manuscript lacks essential information about receptive field sizes, eccentricities, and the number of search stimuli falling within each receptive field and related proper controls. This is critical because receptive fields in visual area V4 at foveal eccentricities are relatively small (Gattass et al., 1988; Desimone & Schein, 1987), whereas receptive fields in inferotemporal cortex can span several degrees to tens of degrees and often include the fovea (Op de Beeck & Vogels, 2000; DiCarlo & Maunsell, 2003; Zoccolan et al., 2007). Given the 2{degree sign} × 2{degree sign} stimulus size, multiple search items could potentially fall simultaneously within peripheral receptive fields. This introduces a potential confound, as attentional modulation is known to be strongest when multiple stimuli appear within a single receptive field (Reynolds et al., 1999). Although the authors acknowledge this issue for visual area V4 (p. 17), it is neither quantified nor controlled for. Without explicit receptive field mapping relative to the search array, comparisons between foveal and peripheral units, as well as between visual areas, are difficult to interpret cleanly.

We thank the reviewer for the helpful suggestion and apologize for not explicitly providing essential information about the RFs of the units. We added a detailed description of RF properties to the Results as follows:

“The RFs of these peripheral units were further mapped using a visually guided saccade task and quantified by the number of stimuli that activated each unit (Fig. 1F-K). The eccentricities of the peripheral RFs were 6.22° ± 1.31° (mean ± SD) in V4, 7.04° ± 1.52° in IT, and 6.68° ± 1.56° in LPFC. The sizes of the peripheral RFs were 3.67° ± 1.87° in V4, 6.86° ± 3.11° in IT, and 8.65° ± 3.02° in LPFC. The numbers of items from the search array falling within peripheral RFs were 1.49 ± 0.55 in V4, 2.2 ± 0.72 in IT, and 2.56 ± 0.74 in LPFC (also see our previous study [44]).”

The reviewer is correct that multiple items from the search array did fall within the RFs of peripheral-RF units. However, for focal foveal units, only the fixated stimulus fell within the RF, due to the design of the search array and the definition of these units used in our analyses (see our reply to Reviewer 1, Public Review, Question 2 for details). We agree with the reviewer that attentional modulation is typically stronger when multiple stimuli fall within RFs. In our design, peripheral RFs, on average, contained more stimuli than foveal RFs. Therefore, this difference in RF size would, if anything, be expected to bias toward stronger attentional modulation in peripheral units. This would make our observation conservative, thereby further supporting rather than undermines our main finding of robust feature-based attentional enhancement in foveal units, challenging the prevailing view that such modulation is predominantly peripheral. However, we agree that, when comparing the latency of attentional effects across brain regions in Fig. 3, we cannot rule out the influence of the number of stimuli arising from differences in RF size.

(2) Attentional modulation is difficult to dissociate from saccade planning and decision-related signals. The free-gaze paradigm enhances ecological validity but introduces a temporal confound: mean distractor fixation durations are approximately 156 ms (p. 9), while attentional effects emerge between 137 and 170 ms after fixation onset (Figure 2). As a result, the reported attentional modulation coincides with the preparation of the subsequent saccade. Neural activity measured in the primary analysis window (150-225 ms; p. 19), therefore, likely reflects a mixture of visual, attentional, motor planning, target recognition, and behavioral relevance signals, all of which are known to modulate responses in visual areas at similar latencies (e.g., Chelazzi et al., 1998). Moreover, target fixations (~257 ms) and distractor fixations (~156 ms) occur on fundamentally different behavioral timescales, which may inflate apparent foveal attentional effects. While the authors suggest that these timing differences support the idea that foveal feature-based attention facilitates prolonged fixation on target stimuli, this interpretation is not fully supported by the current analyses. That said, the saccade-aligned analyses of peripheral units (Figure S3) partially mitigate this concern by demonstrating that featurebased modulation persists through saccade execution.

We thank the reviewer for raising this important question. We agree that the temporal overlap of visual, motor planning, target recognition, and behavioral relevance signals with attention can result in mixed activity, which needs to be dissociated. Therefore, when calculating feature-based attention, we did implement a series of controls. We added a discussion as follows:

“A major challenge in interpreting neural activity related to attentional modulation is the inherent temporal overlap of visual processing, motor planning, and target recognition signals in the free-gaze visual search task [73]. To isolate genuine feature-based attention from potential confounds, we applied several stringent analytical constraints, consistent with prior studies [3, 5, 6]. Specifically, by restricting our analysis to fixations where the subsequent saccade was directed away from the RFs, we dissociated attentional modulation from the preparatory motor activity associated with saccade execution. Furthermore, by comparing responses to the same physical stimulus alternating its role as a target or distractor across trials we eliminated any potential bias introduced by stimulus identity or physical category. We restricted our analysis to fixations preceding target selection that is, before the monkeys made a behavioral choice to minimize contributions from target detection or decision-related signals.”

We thank the reviewer for pointing out the issue of different timescales for target versus distractor fixations. To address this, we conducted a control analysis by computing foveal feature-based attentional modulation using fixations on targets and distractors with matched fixation durations. We obtained similar results. We have updated Fig. S2 to include this control analysis.

We also clarified this point in the Results as follows:

“We also obtained similar results when controlling for fixation durations on targets and distractors (i.e., there was no significant difference between fixation durations on targets and distractors; Wilcoxon signed-rank test, P > 0.05; Fig. S2K–P).”

Lastly, as the reviewer correctly pointed out, the interpretation that foveal feature-based attention facilitates prolonged fixation on the target was not supported. We have revised the Results as follows:

“On average, target fixations (256.69 ± 197.44 ms [mean ± SD]) were significantly longer than distractor fixations (156.26 ± 45.94 ms; Wilcoxon rank-sum test, *P* < 0.0001), and during these prolonged target fixation, foveal feature-based attention modulation was consistently observed.”

(3) The "attention-out" condition for spatial attention lacks directional control. In the spatial attention analyses (Figures 4D-F), the "attention-out" condition appears to include all fixations followed by saccades directed away from the receptive field, regardless of saccade direction. This differs from classic spatial attention designs, which typically use controlled anti-saccades or saccades to fixed locations opposite the receptive field (e.g., Moore & Armstrong, 2003; Gregoriou et al., 2009). Saccades directed toward locations adjacent to, but outside, the receptive field may still partially engage spatial attention mechanisms near the receptive field via broad attentional fields or motor preparation gradients (Bisley & Goldberg, 2010). In addition, the "attention-out" condition likely contains a heterogeneous mixture of trials in which the stimulus in the receptive field is either a target or a distractor, since feature-based attention effects are derived from this same pool of trials. As a result, spatial and feature attention effects are not fully orthogonal, and variance related to feature attention may already be embedded in the spatial attention baseline.

We thank the reviewer for this important question. We performed a directional control analysis by computing spatial attentional modulation using paired fixations from the attention-in and attention-out conditions. Only saccades directed in nearly opposite directions—defined as having a saccade direction angle ≥ 170° within the 0–180° range—were included. We obtained similar results (Author response image 1).

**Author response image 1.** Peripheral spatial attentional modulation in V4, IT, and LPFC. Population response to stimuli followed by saccades directed into their RFs (attention in) versus directed approximately opposite and outside their RFs (attention out), shown for V4 (A), IT (B), and LPFC (C). Shaded area denotes ± SEM across units.

We did control for feature-based attention when calculating spatial attentional modulation. We apologize for the lack of clarity and have added a description of this control to the Methods as follows:

“The saccade-target stimulus in the RF during attention-in fixations was matched to a stimulus in the same location during attention-out fixations; in both conditions, this stimulus always served as a distractor for that trial, except in the “Distractor fixations to T” condition (Fig. 5 and Fig. S4), in which it instead served as the target. This design eliminates differences due to feature-based attention between the attention-in and attention-out conditions.”

**Recommendations for the authors:**

**Reviewer #1 (Recommendations for the authors):**

(1) Figure 3C: Unclear how to compare LPFC vs V4 for foveal units since only data from peripheral LPFC is shown?

We thank the reviewer for pointing out this mistake. In Fig. 3C, we only compared LPFC peripheral units, V4 peripheral units, and V4 foveal units. We have corrected this in the legend of Fig. 3 as follows:

“Shown are cumulative distributions of feature-attention effect latencies, computed from individual foveal face-, house-, and non-selective units in V4 and IT, and from peripheral non-selective units in V4,

IT, and LPFC.”

(2) On page 8, last para: For units with peripheral RFs ... Is this controlled for whether the saccade is to targets or to distractors?

We thank the reviewer for the question. We indeed addressed this concern by separating fixations based on whether the subsequent saccade was directed to a target or a distractor, and by analyzing attention modulation within each condition. Therefore, attention effects were evaluated while holding the saccade destination constant, effectively controlling for potential confounds related to saccade target selection.

(3) Page 9: The authors find that target fixations were longer than distractor fixations and conclude that this supports the idea that foveal feature-based attention increases fixation duration, but this interpretation is pure conjecture, and there is no experimental manipulation presented in this paper that helps to establish this interpretation.

We thank the reviewer for this important comment. We agree that this observation does not, by itself, support our original interpretation, and we have modified it in the Results. Please refer to the last paragraph of our Reply to Question 2 from Reviewer 3 (Public Review).

(4) Data analysis: receptive field. The authors state that visual response to a cue and the stimulus array was assessed during the 0-200 ms window after stimulus onset. However, after the array onset, the animal could saccade within the 200 ms window. How do the authors ensure uniform stimulation during the 0-200 ms window?

We thank the reviewer for this question. The activity of units in V4, IT, and LPFC within the 200 ms window after array onset primarily reflected visual stimulation prior to saccades, because typical saccade latencies were approximately 150–200 ms, and the response onset latencies of these units were around 50 ms.

(5) On page 19, the authors state that to assess feature attention in peripheral RFs, they divided trials into target and distractor fixations. In the former, there was a target in the neuron's RF. This is confusing. I assume target fixations imply fixating on a target, but the authors may mean fixations where a target is in the RF. Please clarify.

We thank the reviewer for pointing out this confusion. In the original manuscript, we intended to sort fixations by whether a target stimulus was located within the unit’s peripheral RF. To avoid further confusion, we have revised the description in the Methods as follows:

“we sorted fixations during the search period, following a procedure similar to that in our previous study [5], into two types: “target” – a target stimulus was located within the unit’s peripheral RF; and “distractor” – the same stimulus appeared in the same peripheral RF location but served as a distractor.”

(6) Figure S1: Are these example units? How many trials? SEM? The sharp rise and no noise are inconsistent; the former suggests minimal smoothing, while the latter suggests lots of smoothing.

We thank the reviewer for these questions. We showed average responses across all units in Fig. S1. On average, there were 941.79 ± 182.56 trials (mean ± SD across sessions). Shaded areas indicate ± SEM across units. The sharp rise reflects the synchronous response of neurons to the stimulus, while the smooth appearance and low noise result from averaging across a very large number of units and trials.

**Reviewer #2 (Recommendations for the authors):**

Major comments:

(1) One weakness of this manuscript is the lack of a rationale for choosing V4, IT, and PFC. Specifically, what are the predictions of the roles of these respective areas in the integration of current and peripheral (future foveal) views? There is a significant literature linking the pre-saccadic peripheral stimulus and the post-saccadic foveal stimulus, suggesting that both spatial and temporal integration occur. However, whether such integration occurs at high or low cortical levels is unknown. By recording from mid-tier (V4) and high-order areas (IT, PFC), the authors have an opportunity to address this question. However, there is no mention of this topic, either in the introduction, results, or discussion. I find this omission surprising. At the very least, it should contribute to experimental design rationale and some discussion.

We thank the reviewer for the suggestion and we modified and added the rationale to the Introduction and a discussion about this integration. Please refer to our Reply to Question 1 from Reviewer 2 (Public Review).

(2) As both behavior and neural recordings are collected, a figure on saccadic patterns would enhance the reader's understanding. Questions that come to mind are: What does a single search trial look like? How many saccades are there per trial? How often is the target identified after 1, 2, 3, etc saccades? What is the average size of a saccade? Although this is not a study of search strategy per se, a modicum of description of the search sequences would provide context on the behavior. I suggest an illustration of one or more sample trials; a summary of saccade behavior would also be helpful for understanding the data in relation to behavioral performance.

We thank the reviewer for this helpful suggestion. We have modified Fig. 1A and its legend to illustrate the saccadic patterns of monkeys during the search, providing an example of a single search trial. Additionally, we have added a description of saccade behavior to the Results and included Table 1, which summarizes eye movement behavior. Please refer to our Reply to Question 2 from Reviewer 2 (Public Review) for further details.

(3) "Consistently, the probability of making a saccade to a peripheral target was higher following distractor fixations (75.22%) than following target fixations (48.44%, or 63.49% after probability calibration; see Methods), indicating the important role of peripheral feature-based attention in guiding eye movements" It should be noted that this target-oriented visual search is fundamentally a top down task. Once the target is found, the reward is obtained; saccades to distractors are not rewarded, so saccades are more likely. So certainly this task design would increase the post-distractor saccades and decrease the number of post-target saccades. Please clarify the behavioral paradigm: once a reward is obtained, does the task continue, or is a new trial initiated?

We apologize for the confusion regarding the behavioral paradigm. We would like to clarify that when the target was found and fixated for 800 ms, the reward was delivered and no further saccades occurred. However, if the target was not fixated for 800 ms, the search could continue. It is worth noting that the target fixations in our analyses were restricted to those occurring during ongoing search behavior, excluding target fixations associated with trial termination and reward delivery. Moreover, we compared the probability of making a saccade to the target, rather than the absolute number of saccades, following these fixations. We have modified the Results for clarification, as follows:

“Two monkeys performed a category-based visual search task, where their objective was to fixate on one of the two search targets that matched the category of the cue (Fig. 1A, B). Specifically, the monkeys were presented with a central fixation point for 400 ms, followed by a cue lasting 500-1300 ms. After a 500 ms delay, a search array appeared with 11 items, including two targets, randomly chosen from 20 possible locations (Fig. 1E). The monkeys had 4000 ms to find one target and maintain fixation on it for 800 ms to earn a juice reward. Fixating on either target completed the trial, and the monkeys did not search for the second target. A new trial began after the reward. It is worth noting that the two target stimuli matched the category of the cue but were different images. The monkeys were required to maintain fixation throughout the cue and delay periods. During search, however, eye movements were unconstrained, and monkeys could revisit each search distractor or target as long as they did not fixate on a target for 800 ms.”

(4) The fact that there are many more peripheral units in LPFC suggests that this is a region of foveal/periph integration. Combined with the finding that the LPFC leads the attentional effects, this should be a discussion point.

We thank the reviewer for the suggestion and we added a discussion as follows:

“Some studies have provided evidence for integration between peripheral and foveal feature information across saccades, including features such as stimulus color [58, 59] and object orientation [60, 61], and visual features have been shown to be predictively remapped prior to saccades [62]. Our finding provides a potential neuronal mechanism that may support this integration process [63]. We found that LPFC’s extensive representation of the visual periphery provides a neural substrate for monitoring the broader search array. Crucially, our finding that LPFC activity temporally precedes attentional effects in the visual area consistent with previous studies [6, 9, 11, 35-40] suggests that it does not merely reflect peripheral sensory input. Instead, LPFC likely acts as a top-down orchestrator, projecting task-relevant templates derived from current foveal goals onto peripheral candidate locations, a possibility that warrants further investigation.”

Minor comments:

(1) Figures 2A-D. "These face-selective units also showed slightly enhanced responses to house targets in IT (P < 0.05), but not in V4 (P = 0.89)." It does not appear enhanced.

We agree with the reviewer that the effect is modest and does not appear strongly enhanced. However, the average response in the 150–225 ms time window to the house target was significantly higher than that to the house distractor in IT face-selective units (Wilcoxon signed-rank test, *P* = 0.042). We modified the description in the Results as follows:

“These face-selective units also showed weakly but significantly enhanced responses to house targets in IT (*P* < 0.05)”

(2) Figure 3. For population comparison, a bootstrapped null distribution was used, and a 2-sided permutation test was used to determine the latency difference between the target and distractor; please show these results (described in text) in a figure. Figures 3A-C are described as the latency of individual units. So each of these graphs is the mean of multiple units? So this is also a population analysis? What is the difference between these two comparisons? This is somewhat confusing.

We apologize for the confusion and thank the reviewer for pointing this out. Each panel in Fig. 3 shows the cumulative distribution of latencies across individual units within each brain region, reflecting the variability of response timing across single neurons. For this analysis, we first calculate the latency of each unit separately. In contrast, population-level latency is measured from the averaged responses of all units within each region (Fig. 2), which captures the overall timing of the population response rather than individual variability. Statistical comparisons at the population level are performed using a two-sided permutation test. We modified Fig. 2 to better illustrate the population-level latency results.

(3) Did peripheral RFs span more than a single stimulus in the array? If so, how does this impact the interpretation of Figure 5?

We thank the reviewer for pointing this out. The reviewer is correct that, in peripheral RFs, more than one stimulus from the search array could fall within the receptive field (1.49 ± 0.55 in V4, 2.2 ± 0.72 in IT, and 2.56 ± 0.74 in LPFC). We controlled for this in our analysis of both feature-based and spatial attention effects for peripheral units in Fig. 5. For feature-based attention, we performed the analysis in a stimulus-by-stimulus manner within each category (house and face), such that when a given stimulus served as the target, it was the only target within the RF, and when it served as a distractor, it was the only distractor of its category within the RF. Although additional distractor could still fall within the RF, their identities were random across conditions and thus would be averaged out. A similar approach was applied to spatial attention, where the stimulus-by-stimulus comparison was extended across all four categories, and attention-out stimuli were paired with the corresponding saccade-target stimuli in the attention-in condition, with the effects of other randomly present distractors averaged out. Therefore, the effects shown in Fig. 5 reflect comparisons at the level of individual stimulus, minimizing confounds from other stimuli within the RF.

(4) Figure 5G: "during "Target fixations to D", there was no significant feature attentional enhancement in response to the peripheral target (Wilcoxon signed-rank test, P > 0.05; Figure 5G-I left panels). It appears that there is some effect of spatial attention during Target Fix to D trials.

We thank the reviewer for pointing this out and have revised the Results as follows:

“We further found that spatial attentional enhancements to the saccade target were reduced during target fixations compared to distractor fixations in V4 and IT when activity was aligned to fixation onset (Wilcoxon rank-sum test, P < 0.05; Fig. 5G, H versus Fig. 5A, B), although this effect was not completely abolished.”

(5) The specific areas of IT and LPFC that were recorded should, as much as possible, be mentioned.

We thank the reviewer for the helpful suggestions and have added a description of the specific IT and LPFC recording sites to the Methods as follows:

“Recordings in IT spanned the central IT cortex, encompassing the area between the anterior middle temporal sulcus (AMTS) and the posterior middle temporal sulcus (PMTS), including TE and TEO. Recordings in LPFC were located anterior to the arcuate sulcus (AS) and lateral to the principal sulcus (PS), mainly covering areas 45 and 44.”

(6) It is often difficult to distinguish the different lines, e.g., red solid vs red dotted, due to their overlap. Would the removal of the error band make this clearer? If so, could put full figure with error bands in the Supplementary Figure.

We thank the reviewer for this helpful suggestion. To improve visual clarity, we adjusted Fig. 6, Fig. 7, Fig. S2, Fig. S3, Fig. S4, and Fig. S6 by changing the line styles and placing the shaded error bands beneath the traces, allowing the lines to remain clearly visible despite overlap.

(7) For easy access, the number of saccades to/from targets/distractors should be put into a table.

We thank the reviewer for the suggestion. We calculated the probability of saccades to and from targets and distractors for each session and report the mean ± SD across sessions in Table 1, as the mean number of saccades per trial was only 2.3. Please refer to our Reply to Question 2 from Reviewer 2 (Public Review) for Table 1.

Reference

(1) O'Craven, K.M., P.E. Downing, and N. Kanwisher, fMRI evidence for objects as the units of attentional selection. Nature, 1999. 401(6753): p. 584-7.

(2) Baldauf, D. and R. Desimone, Neural mechanisms of object-based attention. Science, 2014. 344(6182): p. 424-7.

(3) Hayden, B.Y. and J.L. Gallant, Combined effects of spatial and feature-based attention on responses of V4 neurons. Vision Res, 2009. 49(10): p. 1182-7.

(4) Bichot, N.P., et al., A Source for Feature-Based Attention in the Prefrontal Cortex. Neuron, 2015. 88(4): p. 832-844.

(5) Reddy, L. and N. Kanwisher, Category selectivity in the ventral visual pathway confers robustness to clutter and diverted attention. Curr Biol, 2007. 17(23): p. 2067-72.

(6) Peelen, M.V., L. Fei-Fei, and S. Kastner, Neural mechanisms of rapid natural scene categorization in human visual cortex. Nature, 2009. 460(7251): p. 94-7.

(7) Cukur, T., et al., Attention during natural vision warps semantic representation across the human brain. Nat Neurosci, 2013. 16(6): p. 763-70.

(8) Keller, A.S., et al., Attention enhances category representations across the brain with strengthened residual correlations to ventral temporal cortex. Neuroimage, 2022. 249: p. 118900.

(9) Zhang, J., et al., Behavioral and Neural Mechanisms of Face-Specific Attention during GoalDirected Visual Search. The Journal of Neuroscience, 2024. 44(46): p. e1299242024.

(10) Bichot, N.P., A.F. Rossi, and R. Desimone, Parallel and serial neural mechanisms for visual search in macaque area V4. Science, 2005. 308(5721): p. 529-534.

(11) Bichot, N.P., et al., The role of prefrontal cortex in the control of feature attention in area V4. Nat Commun, 2019. 10(1): p. 5727.

(10) Cohen, M.R. and J.H. Maunsell, Using neuronal populations to study the mechanisms underlying spatial and feature attention. Neuron, 2011. 70(6): p. 1192-204.

(11) Maunsell, J.H. and S. Treue, Feature-based attention in visual cortex. Trends Neurosci, 2006. 29(6): p. 317-22.

(12) McAdams, C.J. and J.H. Maunsell, Attention to both space and feature modulates neuronal responses in macaque area V4. J Neurophysiol, 2000. 83(3): p. 1751-5.

(13) Motter, B.C., Saccadic momentum and attentive control in V4 neurons during visual search. J Vis, 2018. 18(11): p. 16.

(14) Sapountzis, P., S. Paneri, and G.G. Gregoriou, Distinct roles of prefrontal and parietal areas in the encoding of attentional priority. Proc Natl Acad Sci U S A, 2018. 115(37): p. E8755-E8764.

(15) Treue, S. and J.C. Martinez Trujillo, Feature-based attention influences motion processing gain in macaque visual cortex. Nature, 1999. 399(6736): p. 575-9.

(16) Zhou, H. and R. Desimone, Feature-based attention in the frontal eye field and area V4 during visual search. Neuron, 2011. 70(6): p. 1205-17.
